# ggClusterNet 2: An R package for microbial co‐occurrence networks and associated indicator correlation patterns

**DOI:** 10.1002/imt2.70041

**Published:** 2025-04-25

**Authors:** Tao Wen, Yong‐Xin Liu, Lanlan Liu, Guoqing Niu, Zhexu Ding, Xinyang Teng, Jie Ma, Ying Liu, Shengdie Yang, Penghao Xie, Tianjiao Zhang, Lei Wang, Zhanyuan Lu, Qirong Shen, Jun Yuan

**Affiliations:** ^1^ Jiangsu Provincial Key Lab for Organic Solid Waste Utilization, Jiangsu Collaborative Innovation Center for Solid Organic Wastes, Educational Ministry Engineering Center of Resource‐saving Fertilizers, Key Laboratory of Green Intelligent Fertilizer Innovation Nanjing Agricultural University Nanjing China; ^2^ Genome Analysis Laboratory of the Ministry of Agriculture and Rural Affairs, Agricultural Genomics Institute at Shenzhen Chinese Academy of Agricultural Sciences Shenzhen China; ^3^ Inner Mongolia Academy of Agricultural and Animal Husbandry Sciences, Key Laboratory of Black Soil Protection and Utilization (Hohhot), Ministry of Agriculture and Rural Affairs Inner Mongolia Key Laboratory of Degradation Farmland Ecological Restoration and Pollution Control Hohhot China; ^4^ National Agricultural Experimental Station for Agricultural Environment, Luhe Jiangsu Academy of Agricultural Sciences Nanjing China

**Keywords:** microbial co‐occurrence networks, modularity, multi‐omics network, multi‐network comparison, network visualization, transkingdom networks

## Abstract

Since its initial release in 2022, *ggClusterNet* has become a vital tool for microbiome research, enabling microbial co‐occurrence network analysis and visualization in over 300 studies. To address emerging challenges, including multi‐factor experimental designs, multi‐treatment conditions, and multi‐omics data, we present a comprehensive upgrade with four key components: (1) A microbial co‐occurrence network pipeline integrating network computation (Pearson/Spearman/SparCC correlations), visualization, topological characterization of network and node properties, multi‐network comparison with statistical testing, network stability (robustness) analysis, and module identification and analysis; (2) Network mining functions for multi‐factor, multi‐treatment, and spatiotemporal‐scale analysis, including *Facet.Network()* and *module.compare.m.ts()*; (3) Transkingdom network construction using microbiota, multi‐omics, and other relevant data, with diverse visualization layouts such as *MatCorPlot2()* and *cor_link3()*; and (4) Transkingdom and multi‐omics network analysis, including *corBionetwork.st()* and visualization algorithms tailored for complex network exploration, including *model_maptree2()*, *model_Gephi.3()*, and *cir.squ()*. The updates in *ggClusterNet 2* enable researchers to explore complex network interactions, offering a robust, efficient, user‐friendly, reproducible, and visually versatile tool for microbial co‐occurrence networks and indicator correlation patterns. The *ggClusterNet 2*R package is open‐source and available on GitHub (https://github.com/taowenmicro/ggClusterNet).

## Highlights

- The *ggClusterNet 2* introduces a comprehensive microbial co‐occurrence network analysis pipeline.
- Enhanced network analysis workflow tailored for complex experimental designs and diverse data types.
- Enhanced visualization of microbiomes and their correlated environmental or host‐associated indicators.
- Introduced various visualization algorithms for transkingdom and multi‐omics interaction networks.

## Graphical abstract

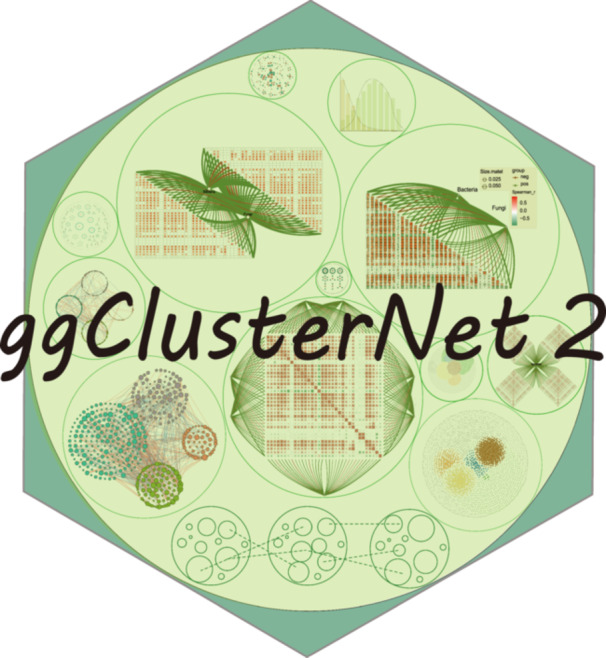

## INTRODUCTION

Microorganisms predominantly exist in complex communities, where cooperative and competitive interactions are crucial in biogeochemical cycles [1, 2], human health [3, 4], animal nutrition [5], and plant stress resistance [6, 7, 8]. Network analysis has become a powerful tool for studying microbial dynamics, and it is widely applied in these fields. For instance, Guidi et al. used microbial networks to show that rare taxa act as “bridges” in oligotrophic marine ecosystems [9]. Similarly, Durán et al. employed transkingdom network analysis of *Arabidopsis thaliana* root‐associated bacteria and fungi, revealing that the negative correlation between *Fusarium* (fungi) and *Pseudomonas* (bacteria) predicts plant pathogen resistance, with network‐guided interventions improving crop yields [10]. Recently, the application of microbial networks has grown increasingly complex, reflecting the expanding scope of microbial ecology. This includes temporal [11] and spatial studies [12] and analyses linking microbial dynamics to host status and phenotypic changes [13, 14]. These complexities require constructing multiple microbial networks and comparing their properties, stability, and modularity [15, 16, 17]. Conventional interactive microbiome analysis tools (e.g., Cytoscape [18], Gephi [19]) struggle to meet these growing demands [20].

Microbiome research increasingly integrates correlated indicators such as environmental factors [21], climate variables [22], and plant growth/stress markers [23] to better elucidate microbe‐host interactions and their downstream effects or upstream effects leading to microbial changes. Additionally, transkingdom interactions are gaining attention for their roles in biogeochemical cycles, human health, and plant resilience. For example, Liu et al. used co‐occurrence networks to explore relationships among protists, antibiotic‐resistance genes, and metazoans [24]. Durán et al. identified intra‐ and inter‐kingdom interaction patterns through transkingdom networks [10], while Sonnert et al. analyzed extensive host‐microbe transkingdom linkages using network analysis [25]. These applications require network analysis tools with enhanced data compatibility, advanced analytics, and flexible visualization.

The *ggClusterNet* R package [26], initially designed for microbiome network analysis, has undergone major updates to keep pace with rapid advancements in microbial network research. These upgrades include: (1) A microbial co‐occurrence network analysis pipeline incorporating network computation and visualization (Pearson, Spearman [27], and SparCC [28] correlations), topological characterization of network and node properties, multi‐network comparison and statistical testing, network stability analysis [29], and network module identification and analysis; (2) Microbial network mining functions for multi‐factor, multi‐treatment, and spatiotemporal analysis, including *Facet.Network()*, *module.compare.m.ts()*, and *Robustness.Random.removal.ts()*; (3) Functions for microbial and multi‐factor interaction analysis, along with versatile visualization algorithms such as *MatCorPlot2()*, *Miccorplot3()*, *cor_link3()*, *matcorplotj()*, and *two.cor()*; and (4) Tools for transkingdom and multi‐omics integrated network analysis, including *corBionetwork.st()*, and a suite of visualization algorithms optimized for complex network relationships, such as *model_maptree2()*, *model_Gephi.3()*, *cir.squ()*, and *cir.maptree2()*.

## RESULTS

### Overview of ggClusterNet 2

**Figure 1:**
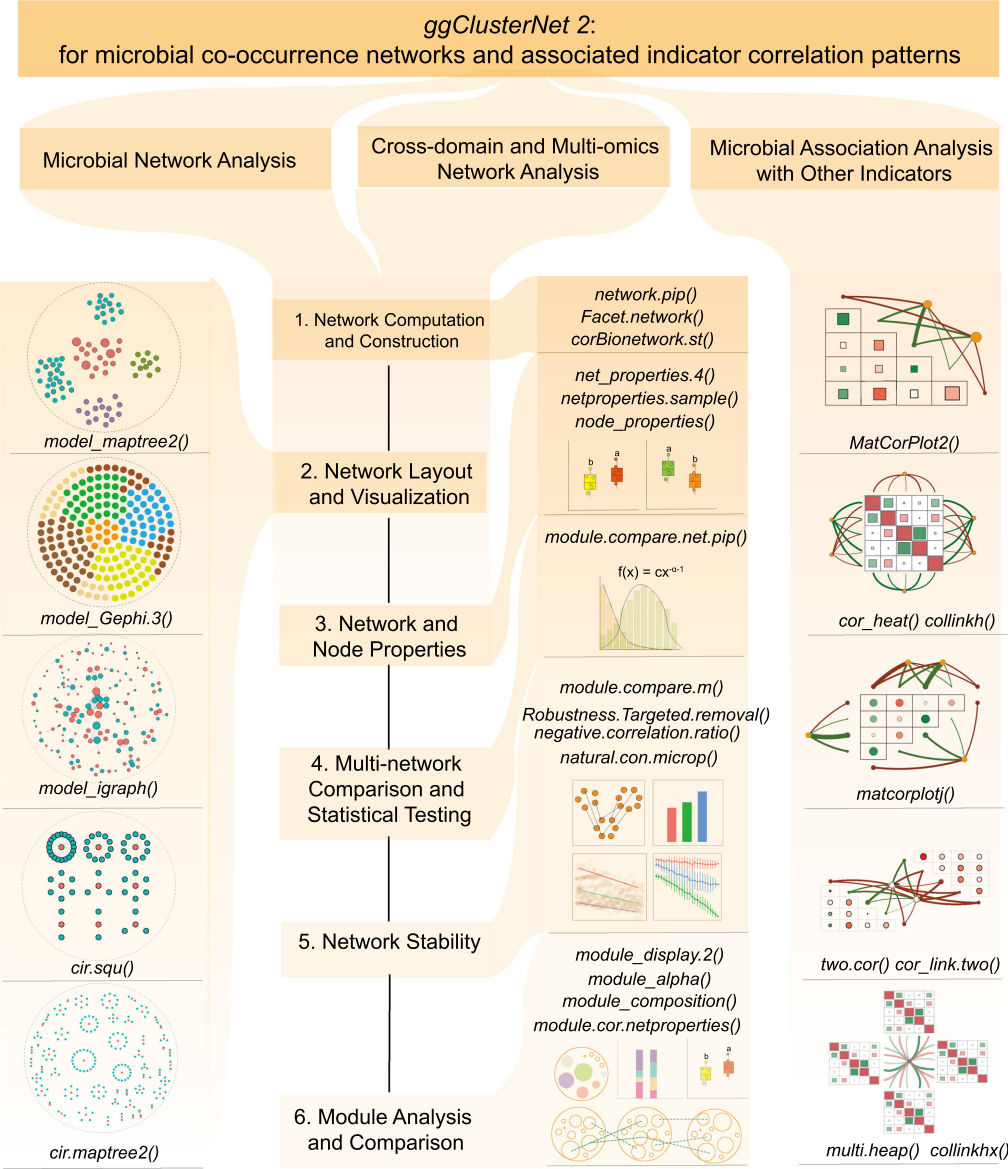
Microbiome network analysis using *ggClusterNet 2*.

We developed an enhanced microbial network analysis workflow in the updated version of the *ggClusterNet 2* package (Figure 1), incorporating microbiome network and transkingdom network analysis workflows, along with the integration of microbiome network data with other indicators. The left panel shows five novel network visualization algorithms optimized to enhance module representation, thereby facilitating the identification of target entities among thousands of variables. The right panel illustrates their effectiveness in analyzing correlations between multi‐omics data and other indicators. By integrating connecting lines and heatmaps, these algorithms reveal potential transkingdom interactions.

### Design and implementation of the microbial network analysis pipeline

**Figure 2:**
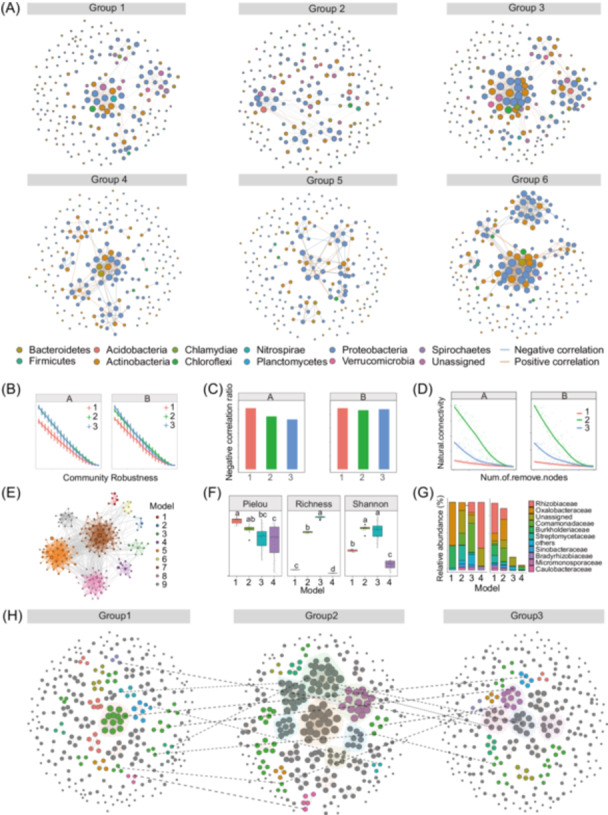
Proposed comprehensive microbiome network analysis pipeline encompassing network construction and visualization, network and node property analysis, multi‐network comparison and statistical assessment, network robustness evaluation, and network module characterization. (A) Visualization of networks across groups using the *model_maptree()* layout algorithm via the *Facet.network()* function. (B) Network stability analysis using the *Robustness.Random.removal.ts()* function. (C) Percentage of negative correlation edges across network groups. (D) Network vulnerability assessment, where the *natural.con.microp.ts()* function indicates network stability. (E) Network module visualization, with nodes in the same module sharing a color. (F) Boxplot of module alpha diversity. (G) Stacked bar plot showing high‐abundance species composition within modules. (H) Visualization of modules across networks, where similar modules share a color, and they are linked by dashed lines.

We introduced a comprehensive upgrade with five key components: (1) network construction and visualization (Figure 2A); (2) network and node property computation; (3) multi‐network comparisons with statistical validation; (4) network stability assessment, including robustness (Figure 2B), negative correlation edge percentage (Figure 2C), and network vulnerability (Figure 2D); and (5) modular analysis and cross‐network module comparisons, including network module display (Figure 2E), composition analysis (Figure 2F), and alpha diversity analysis (Figure 2G). The *network.pip()* function integrates these analyses into a streamlined pipeline by sequentially executing downstream functions (Figure 2E). Additionally, the *Facet.network()* function enhances microbiome network analysis across spatiotemporal scales and complex treatment conditions (Figure 2A). This function supports up to three grouping variables for network visualization, leveraging the faceting concept from *ggplot2* to maintain uniform color, size, and esthetic scaling across numerous network graphs. Multi‐network comparison in network analysis includes evaluating scale‐free properties against random networks and assessing correlations among multiple networks. Specifically, we fit a power‐law distribution to both the constructed and randomized networks (with matching node and edge counts) and compare their fits to assess scale‐free properties. We then used Fisher's test to assess correlations among multiple networks, where *p* < 0.05 indicates significant correlations. This approach improves the efficiency of multi‐network comparisons (Figure 2).

### Transkingdom network analysis of data from microbiome, multi‐omics, and other correlated indicators

**Figure 3:**
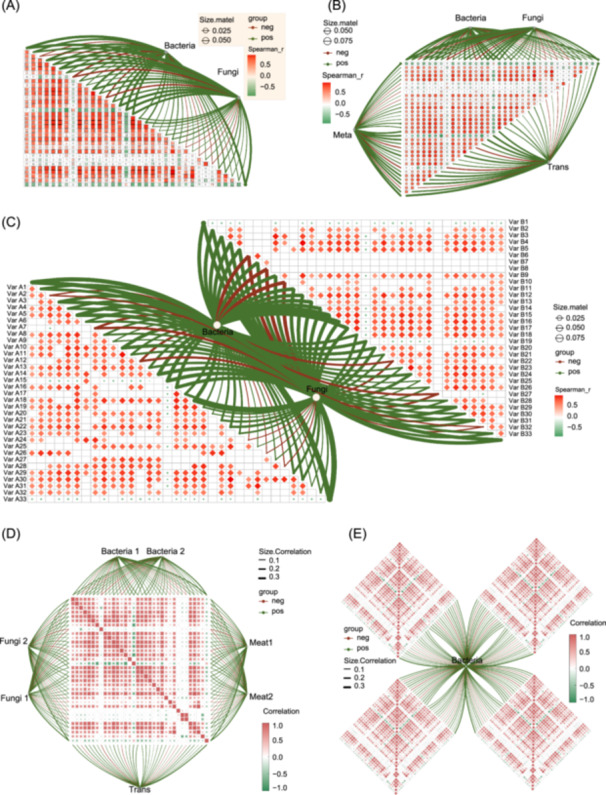
Correlation between multi‐omics data and various indicators. (A) The plot shows the correlation between microbial communities and other indicators. The lower triangular heatmap represents correlations among nonmicrobial indicators, while circular points outside the heatmap denote microbial communities. Connecting lines between these points and the heatmap signify the correlations between microbial communities and other indicators. (B) Correlations between multiple microbial communities or multi‐omics data and other indicators. (C) Correlations between microbial communities and two categories of external indicators. (D) Correlations between multi‐omics data and other indicators. (E) Correlations between microbial communities and four categories of external indicators.

Microbial communities are often analyzed as influencing factors or as variables affected by other conditions. Transkingdom networks integrate microbiome, multi‐omics, and correlated indicators to study microbial‐host interactions and multi‐omics correlations [30, 31, 32, 33]. To systematically analyze these relationships, we developed the *MatCorPlot2()* function, which generates composite plots (Figure 3A) that combine a triangular heatmap (showing correlations among nonmicrobial factors) with a partial heatmap linked to discrete microbial community nodes. To address the growing complexity of microbiome studies, we developed the *matcorplotj()* function, which enables the flexible visualization of microbial correlations in any triangular heatmap direction (Figure 3B). Additionally, to examine the relationships between microbial communities and two categories of external variables (Figure 3C), we designed the *two.cor()* function. The *multi.heap()* and *collinkhx()* functions were also developed to generate multiple heatmaps (Figure 3D,E), facilitating the analysis of microbial community associations with various indicators.

### Transkingdom microbial co‐occurrence network analysis with adaptive visualization frameworks

**Figure 4:**
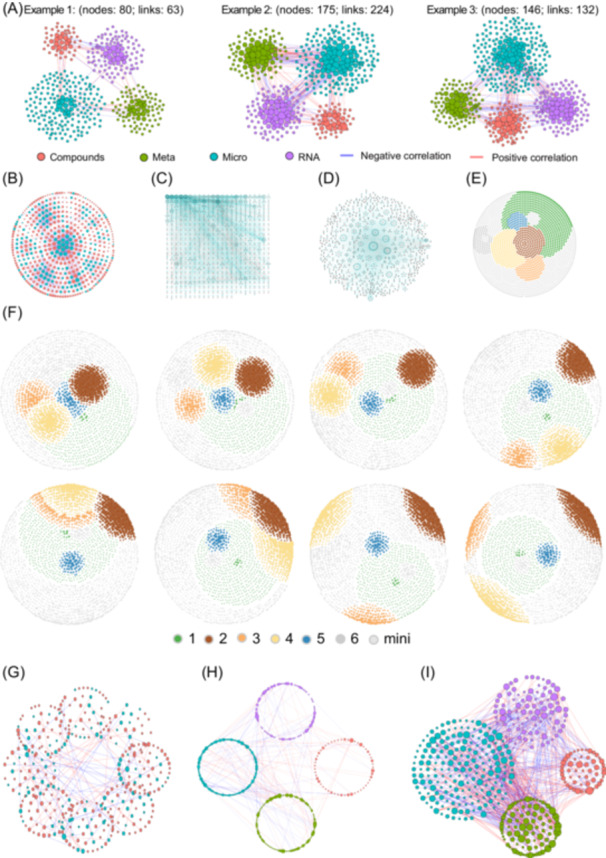
Latest version of *ggClusterNet 2* updates the transkingdom network analysis pipeline, *corBionetwork.st()*, and introduces advanced visualization layout algorithms, including *model_maptree2()*, *model_Gephi.2()*, *cir.squ()*, *cir.maptree2()*, and *model_Gephi.3()*, to improve the representation of transkingdom networks. Furthermore, existing visualization layout algorithms have been optimized and upgraded. (A) Network visualization results produced by the *corBionetwork.st()* function, showcasing the effects across different groups using the *model_maptree()* layout algorithm. (B) Visualization using the *model_Gephi.2()* layout algorithm. (C) Visualization using the *cir.squ()* layout algorithm. (D) Visualization using the *cir.maptree2()* layout algorithm. (E) Visualization using the *model_Gephi.3()* layout algorithm. (F) A series of plots showing the control of module positions through parameter adjustments in the *model_Gephi.3()* algorithm. (G) Display of transkingdom networks using the *PolygonClusterG()* layout algorithm. (H) Display of transkingdom networks using the *PolygonRrClusterG()* layout algorithm. (I) Display of transkingdom networks using the *model_filled_circle()* layout algorithm.

To address the increasing demand for cross‐domain microbial co‐occurrence network analysis and integrated multi‐omics network analysis, we developed the *corBionetwork.st()* function (Figure 4A). This function efficiently handles diverse data types while ensuring compatibility with spatiotemporal research designs, facilitating comprehensive microbial network analysis and comparison. The latest version of *ggClusterNet 2* introduces advanced visualization layout algorithms, including *model_maptree2()* (Figure 4A), *model_Gephi.2()* (Figure 4B), *cir.squ()* (Figure 4C), *cir.maptree2()* (Figure 4D), and *model_Gephi.3()* (Figure 4E), to enhance the visualization of transkingdom networks. The *model_maptree2()* algorithm optimizes module positioning based on network modularity, considering the relative positions of modules to show inter‐module relationships more accurately. model_Gephi.3*()* enables continuous dynamic adjustment of multi‐module relative positions through parameter tuning, allowing customized network layouts (Figure 4F). The *cir.squ()* and *cir.maptree2()* layouts specialize in identifying hub nodes within network modules. Furthermore, the updated version includes the *calculate_node_axis()* function to optimize and invoke these layout algorithms, such as *PolygonClusterG()* (Figure 4G), *PolygonRrClusterG()* (Figure 4H), and *model_filled_circle()* (Figure 4I), further improving the interpretation of critical information in transkingdom networks.

## DISCUSSION

Network analysis has been a key tool in microbiome research and related fields for over a decade, especially with the rise of third‐generation sequencing technologies, such as Oxford Nanopore [34, 35, 36]. The scope of microbial network analysis has evolved from basic correlation calculations and visualization to more advanced analyses, including network and node property profiling, module identification, and assessments of network stability and robustness [37]. Building on these advancements, we have systematically reviewed the methodologies and developed an integrated workflow specifically tailored for modern microbial network research. This workflow includes network construction and visualization, quantification of network and node properties, multi‐network structure comparison with statistical testing, exploration of network stability (robustness), and module identification analysis. By facilitating accurate, efficient, and reproducible network analyses, this workflow enhances the rigor and applicability of microbial network studies.

After developing and releasing this workflow as open‐source on GitHub, we conducted an extended public testing phase to evaluate its reliability and usability. During this phase, multiple researchers validated the workflow across diverse research applications. For example, Ma et al. employed the new *network.pip()* function to analyze the interaction mechanisms between *Bacillus* and *Mucor‐*enhanced biofertilizers in shaping soil microbial communities [38]. Similarly, Niu et al. showed that stimulating nonantibiotic resistance gene‐harboring microbes with specific carbon sources can suppress soil antibiotic‐resistant bacteria rebound after calcium peroxide treatment [39]. Additionally, Hu et al. used a new network visualization layout to establish links between grassland degradation‐induced soil organic carbon loss and simplified micro‐food webs [40]. Based on insights from public testing, we refined the network stability calculation, module detection, and visualization layout functions in this update to better, ensuring enhanced alignment with the evolving demands of microbiome research.

The application of network analysis in scientific research has become increasingly complex, driven by the rising demands and challenges of modern studies. Additionally, microbial network construction often incorporates additional correlated indicators, such as evaluating the effects of environmental factors on microbial communities [41] and understanding dynamic changes in microbe‐plant interactions [42, 43]. Simultaneously, microbial network analysis plays a critical role in exploring bacterial‐fungal interactions [44] and conducting multi‐omics analyses [15, 45]. To address these demands, *ggClusterNet* has been developed with advanced microbial‐multimeric correlation analysis and visualization capabilities, enabling the efficient execution of complex analyses. Furthermore, to facilitate the visualization of correlations between various metrics and multiple microbial communities, we enhanced functions such as *MatCorPlot2()*, *Miccorplot3()*, and *cor_link()*. Microbiome studies increasingly emphasize the effect of multifactorial factors on microbial dynamics, highlighting the need for robust and versatile analytical tools. For instance, Vujkovic‐Cvijin et al. emphasize the confounding effects of multifactorial variables (e.g., age, body mass index, and medication) on disease‐associated microbiome research [46]. Similarly, Wilmanski et al. integrated factors such as age, diet, and metabolism to explore the relationship between the microbiome and healthy aging [47]. These studies require efficient comparison of multiple networks on a unified scale, a challenge that existing tools still struggle to address. Liu et al. released a guide for comparing microbial co‐occurrence networks, providing detailed comparisons of attributes, modules, and characteristic nodes across multiple networks [17]. However, the guide lacks coverage of network stability and visualization layouts for multi‐network comparison. To address this, we developed the *Facet.network()* and *corBionetwork.st()* functions, which incorporate temporal and spatial variables to facilitate visualization and comparison using consistent color and size scales. Additionally, the upgraded *network.pip()* function enhances module similarity analysis and visualization across networks, significantly improving efficiency and analytical scope.

Transkingdom networks have recently gained prominence in studying cross‐boundary microbial interactions and multi‐omics correlations, underscoring the need for robust analytical frameworks. This demand has driven advancements in network analysis, multi‐network comparison, module identification, and advanced visualization layout algorithms. In this update, we developed visualization layout algorithms, including *model_maptree2()*, *model_Gephi.3()*, *cir.squ()*, and *cir.maptree2()*, to enhance these capabilities. For instance, the *cir.squ()* algorithm effectively identifies key functional modules and core nodes, organizing them into rectangular layouts. Concurrently, *cir.maptree()* clusters small modules and optimizes the spatial arrangement of larger modules, incorporating relative positional relationships between modules to enhance inter‐module association analysis and overall network clarity. For instance, Banerjee et al. used a similar approach to investigate how environmental factors influence relationships by affecting submodules [48]. However, their method required manual adjustments in Cytoscape, limiting its applicability to complex multi‐network comparisons and transkingdom interactions. The latest version of *ggClusterNet* addresses these challenges by offering automated, scalable solutions that enhance analytical efficiency and depth.

## CONCLUSION

The *ggClusterNet 2* update comprehensively addresses current research demands in microbiome and related indicator network analysis. It introduces an extensive set of network analysis functions, enhancing convenience for researchers using networks to explore relevant research questions. *ggClusterNet 2* actively responds to the increasing demand for network analysis in multi‐factor, multi‐treatment, transkingdom interactions, and multi‐omics integrative studies. The update introduces new analytical functions and optimizes existing ones to keep pace with rapid advancements in related research fields. Additionally, the update significantly improves network visualization algorithms, offering new layouts, such as *model_maptree2*, *model_Gephi.3*, *cir.squ*, and *cir.maptree2*, while enhancing the practicality and efficiency of existing layouts. *ggClusterNet 2* is also compatible with major microbiome analysis pipelines, such as QIIME 2 [49] and EasyAmplicon [50]. Overall, *ggClusterNet 2* drives the evolution of network analysis, offering researchers an accurate, efficient, convenient, reproducible, and visually compelling tool. We aim to deliver a network analysis solution that meets the highest standards of clarity and usability.

## AUTHOR CONTRIBUTIONS

**Tao Wen**: Methodology; writing—original draft; writing—review and editing; data curation; resources; funding acquisition. **Yong‐Xin Liu**: Conceptualization; writing—review and editing; methodology. **Lanlan Liu**: Validation; writing—original draft; investigation. **Guoqing Niu**: Validation; writing—original draft. **Zhexu Ding**: Validation; writing—original draft. **Xinyang Teng**: Validation; writing—original draft. **Jie Ma**: validation; writing—original draft. **Ying Liu**: Validation; writing—original draft. **Shengdie Yang**: Validation; writing—original draft. **Penghao Xie**: Validation; writing—original draft. **Tianjiao Zhang**: Validation; writing—original draft. **Lei Wang**: Validation; writing—original draft. **Zhanyuan Lu**: Funding acquisition; resources; supervision; writing—review and editing. **Qirong Shen**: Writing—review and editing; supervision. **Jun Yuan**: Supervision; resources; writing—review and editing; funding acquisition.

## CONFLICT OF INTEREST STATEMENT

The authors declare no conflicts of interest. Yongxin Liu serves as the Executive Editor of iMeta.

## ETHICS STATEMENT

No ethics approval was required for this study because all samples used were publicly available and obtained from open‐access sources.

## Data Availability

The data that support the findings of this study are openly available in ggClusterNet at https://github.com/taowenmicro/ggClusterNet, reference number v.2.00. *ggClusterNet 2* is publicly accessible to all researchers in GitHub (https://github.com/taowenmicro/ggClusterNet/). The data and scripts used are saved in GitHub (https://github.com/taowenmicro/ggClusterNet/wiki). Supplementary materials (graphical abstract, slides, videos, Chinese translated version and update materials) may be found in the online DOI or iMeta Science http://www.imeta.science/.
